# *N*′-[(*E*)-4-(Diethyl­amino)benzyl­idene]-4-nitro­benzohydrazide monohydrate

**DOI:** 10.1107/S1600536810011554

**Published:** 2010-03-31

**Authors:** Tanveer Ahmad, Muhammad Zia-ur-Rehman, Hamid Latif Siddiqui, Syed Umar Farooq Rizvi, Masood Parvez

**Affiliations:** a Institute of Chemistry, University of the Punjab, Lahore 54590, Pakistan; b Applied Chemistry Research Centre, PCSIR Laboratories Complex, Lahore 54600, Pakistan; c Department of Chemistry, The University of Calgary, 2500 University Drive NW, Calgary, Alberta, Canada T2N 1N4

## Abstract

In the title compound, C_18_H_20_N_4_O_3_·H_2_O, the two aromatic rings are linked through a methyl­idenehydrazide fragment, which is fully extended with C—C—N—N, C—N—N=C and N—N=C—C torsion angles of 179.4 (2), 174.7 (2) and 178.3 (2)°, respectively. The dihedral angle between the two aromatic rings is 7.01 (8)°. In the crystal structure, the water of hydration is involved in extensive hydrogen bonding. Inter­molecular O—H⋯O, N—H⋯O and O—H⋯N hydrogen bonds link the components of the structure into a two-dimensional network and additional stabilization is provided by weak inter­molecular C—H⋯O hydrogen bonds.

## Related literature

For the synthesis of related compounds, see: Ahmad *et al.* (2010 ▶). For the coordinating capability of hydrazones, see: Rodríguez-Argüelles *et al.* (2004 ▶). For the biological activity of benzohydrazides, see: Zia-ur-Rehman *et al.* (2009 ▶); Galal *et al.* (2009 ▶); Bordoloi *et al.* (2009 ▶). For closely related structures, see: Fun *et al.* (2008 ▶); Bessy *et al.* (2006 ▶).
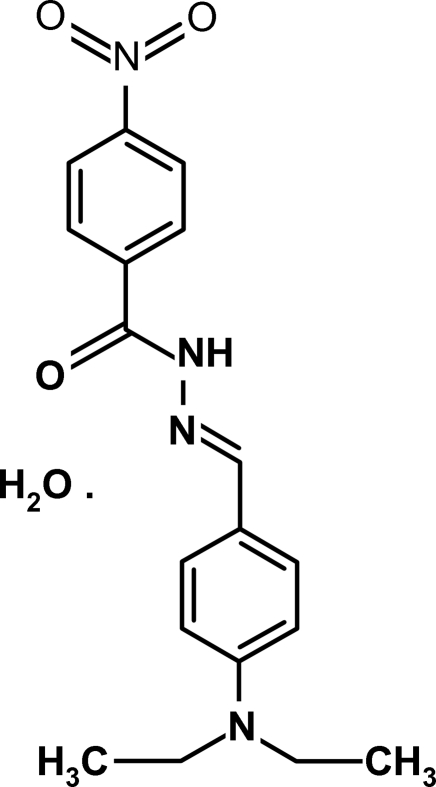


## Experimental

### 

#### Crystal data

C_18_H_20_N_4_O_3_·H_2_O
*M* *_r_* = 358.40
Monoclinic, 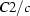

*a* = 38.3142 (12) Å
*b* = 7.4563 (3) Å
*c* = 12.6332 (5) Å
β = 98.215 (2)°
*V* = 3572.0 (2) Å^3^
*Z* = 8
Mo *K*α radiation
μ = 0.10 mm^−1^
*T* = 173 K
0.08 × 0.06 × 0.04 mm

#### Data collection

Nonius KappaCCD diffractometer
Absorption correction: multi-scan (*SORTAV*; Blessing, 1997 ▶) *T*_min_ = 0.992, *T*_max_ = 0.996
5710 measured reflections
4048 independent reflections
3182 reflections with *I* > 2σ(*I*)
*R*_int_ = 0.021

#### Refinement

*R*[*F* ^2^ > 2σ(*F* ^2^)] = 0.059
*wR*(*F* ^2^) = 0.138
*S* = 1.11
4048 reflections
246 parameters
H atoms treated by a mixture of independent and constrained refinement
Δρ_max_ = 0.29 e Å^−3^
Δρ_min_ = −0.19 e Å^−3^

### 

Data collection: *COLLECT* (Hooft, 1998 ▶); cell refinement: *DENZO* (Otwinowski & Minor, 1997 ▶); data reduction: *SCALEPACK* (Otwinowski & Minor, 1997 ▶); program(s) used to solve structure: *SHELXS97* (Sheldrick, 2008 ▶); program(s) used to refine structure: *SHELXL97* (Sheldrick, 2008 ▶); molecular graphics: *ORTEP-3 for Windows* (Farrugia, 1997 ▶); software used to prepare material for publication: *SHELXL97*.

## Supplementary Material

Crystal structure: contains datablocks global, I. DOI: 10.1107/S1600536810011554/bt5231sup1.cif

Structure factors: contains datablocks I. DOI: 10.1107/S1600536810011554/bt5231Isup2.hkl

Additional supplementary materials:  crystallographic information; 3D view; checkCIF report

## Figures and Tables

**Table 1:** Hydrogen-bond geometry (Å, °)

*D*—H⋯*A*	*D*—H	H⋯*A*	*D*⋯*A*	*D*—H⋯*A*
O4—H4*B*⋯O3^i^	0.83 (4)	2.23 (4)	3.009 (3)	156 (3)
N2—H2*N*⋯O4	0.88 (2)	2.00 (2)	2.861 (2)	165 (2)
O4—H4*A*⋯O3^ii^	0.85 (3)	2.06 (4)	2.823 (2)	149 (3)
O4—H4*A*⋯N3^ii^	0.85 (3)	2.57 (3)	3.250 (3)	138 (3)
C5—H5⋯O3^ii^	0.95	2.54	3.308 (2)	138
C8—H8⋯O4	0.95	2.50	3.270 (3)	138
C13—H13⋯O2^iii^	0.95	2.51	3.350 (3)	148

Symmetry codes: (i) 
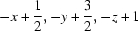
; (ii) 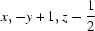; (iii) 
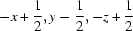
.

**Fig. 1.**
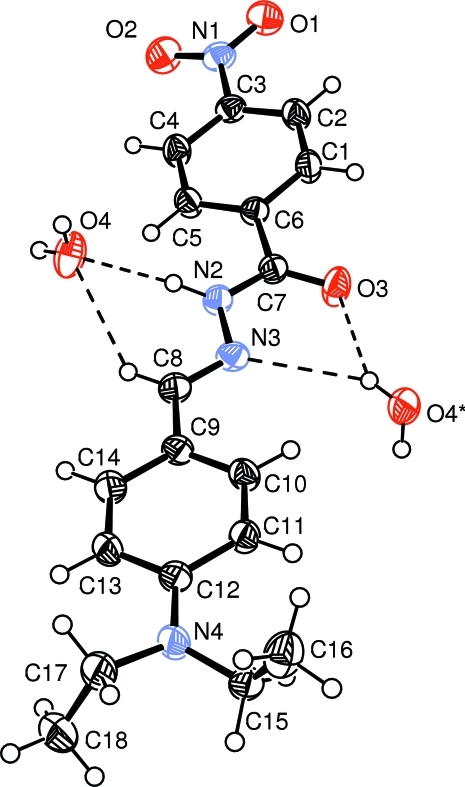
The title compound with the displacement ellipsoids plotted at 50% probability level (Farrugia, 1997). Symmetry code * = x, 1-y, z+1/2.

**Fig. 2.**
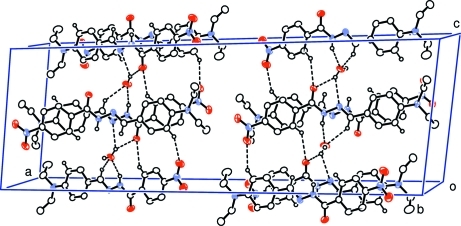
The unit cell packing of the title compound; H-bonds have been plotted with dashed lines and H-atoms not involved in H-bonds have been excluded for clarity.

## supplementary crystallographic information

### Comment

The chemistry of hydrazones is being investigated continuously due to their excellent coordinating capability (Rodríguez-Argüelles *et al.*, 2004) and biological activities (Zia-ur-Rehman *et al.*, 2009; Galal *et al.*, 2009; Bordoloi *et al.*, 2009). In continuation of our studies on the synthesis of various heterocyclic compounds (Ahmad *et al.*, 2010), the title compound, (I), has been synthesized and its crystal structure determined by X-ray crystallographic method which is presented in this article.

In the the title compound (Fig. 1) the bond distances and angles agree with the corresponding bond distances and angles reported in closely related compounds (Fun *et al.*, 2008; Bessy *et al.*, 2006). The benzene rings in (I) are linked through a methylidenehydrazide fragment, C6/C7/N2/N3/C8, which is fully extended with torsion angles C6–C7–N2–N3, C7–N2–N3\ C8 and N2–N3═C8–C9 179.4 (2), 174.7 (2) and 178.3 (2)°, respectively. The dihedral angle between the two benzene rings is 7.01 (8)°. In the crystal structure, the water of hydration is extensively involved in hydrogen bonding. Thus, intermolecular O—H···O, N—H···O and O—H···N hydrogen bonds link the components of the structure into a two-dimensional network and additional stabilization is provided by weak intermolecular C—H···O hydrogen bonds; details have been provided in Table. 1. and Fig. 2.

### Experimental

A mixture of para nitrobenzohydrazide (0.5 g, 2.76 mmoles), *p*-(diethylamino)benzaldehyde (0.49 g, 2.76 mmoles), orthophosphoric acid (0.2 ml) and methanol (50.0 ml) was refluxed for a period of 5.5 hours followed by removal of the solvent under vacuum. The contents were cooled and washed with cold methanol followed by crystallization from the same solvent at room temperature by slow evaporation. Yield: 91%. M.p. 491 K.

### Refinement

Though all the H atoms could be distinguished in the difference Fourier map the H-atoms bonded to C-atoms were included at geometrically idealized positions and refined in riding-model approximation with C—H = 0.95, 0.98 and 0.99 Å, for aryl, methyl and methylene H-atoms, respectively; the coordinates of the H-atoms bonded to N2 and O4   were allowed to refine. The *U*_iso_(H) were allowed at 1.5*U*_eq_(methyl-C and water-O) and 1.2*U*_eq_(the rest of the parent atoms). The final difference map was essentially featurless.

### Crystal data

**Table:**

C_18_H_20_N_4_O_3_·H_2_O	*F*(000) = 1520
*M**_r_* = 358.40	*D*_x_ = 1.333 Mg m^−^^3^
Monoclinic, *C*2/*c*	Melting point: 491 K
Hall symbol: -C 2yc	Mo *K*α radiation, λ = 0.71073 Å
*a* = 38.3142 (12) Å	Cell parameters from 3855 reflections
*b* = 7.4563 (3) Å	θ = 1.0–27.5°
*c* = 12.6332 (5) Å	µ = 0.10 mm^−^^1^
β = 98.215 (2)°	*T* = 173 K
*V* = 3572.0 (2) Å^3^	Prism, orange
*Z* = 8	0.08 × 0.06 × 0.04 mm

### Data collection

**Table:**

Nonius KappaCCD diffractometer	4048 independent reflections
Radiation source: fine-focus sealed tube	3182 reflections with *I* > 2σ(*I*)
graphite	*R*_int_ = 0.021
ω and φ scans	θ_max_ = 27.5°, θ_min_ = 2.1°
Absorption correction: multi-scan (*SORTAV*; Blessing, 1997)	*h* = −49→49
*T*_min_ = 0.992, *T*_max_ = 0.996	*k* = −6→9
5710 measured reflections	*l* = −16→16

### Refinement

**Table:**

Refinement on *F*^2^	Primary atom site location: structure-invariant direct methods
Least-squares matrix: full	Secondary atom site location: difference Fourier map
*R*[*F*^2^ > 2σ(*F*^2^)] = 0.059	Hydrogen site location: difference Fourier map
*wR*(*F*^2^) = 0.138	H atoms treated by a mixture of independent and constrained refinement
*S* = 1.11	*w* = 1/[σ^2^(*F*_o_^2^) + (0.0299*P*)^2^ + 6.8806*P*] where *P* = (*F*_o_^2^ + 2*F*_c_^2^)/3
4048 reflections	(Δ/σ)_max_ < 0.001
246 parameters	Δρ_max_ = 0.29 e Å^−^^3^
0 restraints	Δρ_min_ = −0.19 e Å^−^^3^

### Special details

**Table:**

Geometry. All esds (except the esd in the dihedral angle between two l.s. planes) are estimated using the full covariance matrix. The cell esds are taken into account individually in the estimation of esds in distances, angles and torsion angles; correlations between esds in cell parameters are only used when they are defined by crystal symmetry. An approximate (isotropic) treatment of cell esds is used for estimating esds involving l.s. planes.
Refinement. Refinement of *F*^2^ against ALL reflections. The weighted *R*-factor wR and goodness of fit *S* are based on *F*^2^, conventional *R*-factors *R* are based on *F*, with *F* set to zero for negative *F*^2^. The threshold expression of *F*^2^ > σ(*F*^2^) is used only for calculating *R*-factors(gt) etc. and is not relevant to the choice of reflections for refinement. *R*-factors based on *F*^2^ are statistically about twice as large as those based on *F*, and *R*- factors based on ALL data will be even larger.

### Fractional atomic coordinates and isotropic or equivalent isotropic displacement parameters (Å^2^)

**Table:**

	*x*	*y*	*z*	*U*_iso_*/*U*_eq_	
O4	0.24370 (5)	0.6548 (3)	0.29158 (14)	0.0491 (5)	
H4A	0.2524 (8)	0.625 (5)	0.236 (3)	0.074*	
H4B	0.2376 (9)	0.762 (5)	0.289 (3)	0.074*	
O1	0.44265 (4)	0.7990 (3)	0.48843 (14)	0.0504 (5)	
O2	0.42219 (4)	0.7098 (3)	0.32907 (14)	0.0529 (5)	
O3	0.29243 (4)	0.4979 (3)	0.66984 (12)	0.0470 (5)	
N1	0.41920 (5)	0.7349 (3)	0.42338 (15)	0.0382 (4)	
N2	0.26157 (4)	0.5178 (2)	0.50392 (14)	0.0309 (4)	
H2N	0.2601 (6)	0.555 (3)	0.4374 (19)	0.037*	
N3	0.23060 (4)	0.4687 (2)	0.54236 (14)	0.0329 (4)	
N4	0.06636 (4)	0.2626 (3)	0.54414 (14)	0.0385 (5)	
C1	0.35196 (5)	0.6469 (3)	0.60129 (16)	0.0360 (5)	
H1	0.3497	0.6546	0.6750	0.043*	
C2	0.38313 (5)	0.6981 (3)	0.56723 (17)	0.0376 (5)	
H2	0.4023	0.7423	0.6165	0.045*	
C3	0.38577 (5)	0.6834 (3)	0.45998 (16)	0.0317 (4)	
C4	0.35855 (5)	0.6218 (3)	0.38607 (16)	0.0355 (5)	
H4	0.3611	0.6134	0.3125	0.043*	
C5	0.32730 (5)	0.5720 (3)	0.42139 (16)	0.0331 (5)	
H5	0.3081	0.5294	0.3716	0.040*	
C6	0.32385 (5)	0.5841 (3)	0.52888 (15)	0.0286 (4)	
C7	0.29128 (5)	0.5290 (3)	0.57364 (16)	0.0308 (4)	
C8	0.20420 (5)	0.4474 (3)	0.46932 (17)	0.0330 (4)	
H8	0.2075	0.4627	0.3968	0.040*	
C9	0.16922 (5)	0.4008 (3)	0.49284 (16)	0.0313 (4)	
C10	0.16034 (5)	0.3932 (3)	0.59596 (16)	0.0324 (5)	
H10	0.1778	0.4189	0.6553	0.039*	
C11	0.12662 (5)	0.3491 (3)	0.61358 (16)	0.0327 (5)	
H11	0.1213	0.3454	0.6847	0.039*	
C12	0.09998 (5)	0.3094 (3)	0.52795 (16)	0.0326 (5)	
C13	0.10887 (5)	0.3192 (3)	0.42407 (17)	0.0371 (5)	
H13	0.0915	0.2945	0.3644	0.044*	
C14	0.14265 (6)	0.3645 (3)	0.40804 (17)	0.0373 (5)	
H14	0.1480	0.3711	0.3370	0.045*	
C15	0.05774 (6)	0.2167 (3)	0.64950 (17)	0.0387 (5)	
H15A	0.0788	0.1621	0.6922	0.046*	
H15B	0.0388	0.1253	0.6410	0.046*	
C16	0.04582 (6)	0.3745 (4)	0.7111 (2)	0.0502 (6)	
H16A	0.0392	0.3322	0.7789	0.075*	
H16B	0.0255	0.4321	0.6686	0.075*	
H16C	0.0651	0.4613	0.7256	0.075*	
C17	0.03658 (6)	0.2743 (4)	0.45771 (18)	0.0449 (6)	
H17A	0.0415	0.3697	0.4073	0.054*	
H17B	0.0152	0.3095	0.4884	0.054*	
C18	0.02924 (7)	0.1007 (4)	0.3964 (2)	0.0595 (8)	
H18A	0.0493	0.0714	0.3595	0.089*	
H18B	0.0080	0.1144	0.3438	0.089*	
H18C	0.0257	0.0040	0.4463	0.089*	

### Atomic displacement parameters (Å^2^)

**Table:**

	*U*^11^	*U*^22^	*U*^33^	*U*^12^	*U*^13^	*U*^23^
O4	0.0535 (10)	0.0650 (12)	0.0311 (8)	0.0156 (9)	0.0143 (7)	0.0043 (9)
O1	0.0315 (8)	0.0661 (12)	0.0534 (10)	−0.0138 (8)	0.0055 (7)	0.0024 (9)
O2	0.0483 (10)	0.0704 (13)	0.0443 (9)	−0.0106 (9)	0.0216 (8)	0.0001 (9)
O3	0.0312 (8)	0.0813 (13)	0.0286 (7)	−0.0081 (8)	0.0043 (6)	0.0116 (8)
N1	0.0316 (9)	0.0399 (11)	0.0443 (10)	−0.0039 (8)	0.0098 (8)	0.0046 (9)
N2	0.0241 (8)	0.0398 (10)	0.0297 (8)	−0.0028 (7)	0.0063 (7)	0.0027 (8)
N3	0.0241 (8)	0.0379 (10)	0.0376 (9)	−0.0030 (7)	0.0072 (7)	0.0018 (8)
N4	0.0248 (8)	0.0575 (13)	0.0325 (9)	−0.0085 (8)	0.0011 (7)	0.0024 (9)
C1	0.0307 (10)	0.0519 (14)	0.0248 (9)	−0.0055 (10)	0.0017 (8)	0.0008 (9)
C2	0.0286 (10)	0.0512 (14)	0.0317 (10)	−0.0083 (10)	0.0002 (8)	−0.0005 (10)
C3	0.0261 (9)	0.0335 (11)	0.0359 (10)	−0.0018 (8)	0.0062 (8)	0.0034 (9)
C4	0.0333 (11)	0.0469 (13)	0.0270 (10)	−0.0019 (9)	0.0071 (8)	−0.0027 (9)
C5	0.0282 (10)	0.0419 (12)	0.0287 (10)	−0.0041 (9)	0.0023 (8)	−0.0033 (9)
C6	0.0258 (9)	0.0313 (10)	0.0290 (9)	−0.0006 (8)	0.0049 (8)	0.0010 (8)
C7	0.0287 (10)	0.0354 (11)	0.0289 (10)	−0.0029 (8)	0.0061 (8)	0.0009 (8)
C8	0.0294 (10)	0.0361 (11)	0.0343 (10)	−0.0021 (9)	0.0075 (8)	0.0009 (9)
C9	0.0249 (9)	0.0347 (11)	0.0340 (10)	−0.0024 (8)	0.0028 (8)	0.0010 (9)
C10	0.0258 (9)	0.0377 (11)	0.0322 (10)	−0.0012 (8)	−0.0006 (8)	−0.0009 (9)
C11	0.0263 (10)	0.0415 (12)	0.0299 (10)	−0.0007 (9)	0.0027 (8)	0.0032 (9)
C12	0.0257 (9)	0.0379 (11)	0.0339 (10)	−0.0035 (8)	0.0028 (8)	0.0025 (9)
C13	0.0291 (10)	0.0489 (13)	0.0314 (10)	−0.0071 (9)	−0.0023 (8)	0.0022 (10)
C14	0.0338 (11)	0.0466 (13)	0.0314 (10)	−0.0067 (10)	0.0047 (9)	−0.0001 (9)
C15	0.0280 (10)	0.0515 (14)	0.0363 (11)	−0.0080 (10)	0.0039 (8)	0.0067 (10)
C16	0.0405 (13)	0.0680 (18)	0.0436 (13)	0.0061 (12)	0.0114 (11)	0.0064 (13)
C17	0.0287 (10)	0.0637 (16)	0.0407 (12)	−0.0069 (11)	−0.0006 (9)	0.0075 (11)
C18	0.0498 (15)	0.080 (2)	0.0464 (14)	−0.0243 (14)	−0.0024 (12)	−0.0023 (14)

### Geometric parameters (Å, °)

**Table:**

O4—H4A	0.85 (3)	C8—C9	1.456 (3)
O4—H4B	0.83 (4)	C8—H8	0.9500
O1—N1	1.225 (2)	C9—C10	1.394 (3)
O2—N1	1.228 (2)	C9—C14	1.395 (3)
O3—C7	1.232 (2)	C10—C11	1.382 (3)
N1—C3	1.473 (3)	C10—H10	0.9500
N2—C7	1.339 (2)	C11—C12	1.409 (3)
N2—N3	1.394 (2)	C11—H11	0.9500
N2—H2N	0.88 (2)	C12—C13	1.404 (3)
N3—C8	1.278 (3)	C13—C14	1.380 (3)
N4—C12	1.378 (3)	C13—H13	0.9500
N4—C15	1.457 (3)	C14—H14	0.9500
N4—C17	1.465 (3)	C15—C16	1.517 (4)
C1—C2	1.380 (3)	C15—H15A	0.9900
C1—C6	1.391 (3)	C15—H15B	0.9900
C1—H1	0.9500	C16—H16A	0.9800
C2—C3	1.377 (3)	C16—H16B	0.9800
C2—H2	0.9500	C16—H16C	0.9800
C3—C4	1.376 (3)	C17—C18	1.514 (4)
C4—C5	1.387 (3)	C17—H17A	0.9900
C4—H4	0.9500	C17—H17B	0.9900
C5—C6	1.386 (3)	C18—H18A	0.9800
C5—H5	0.9500	C18—H18B	0.9800
C6—C7	1.499 (3)	C18—H18C	0.9800
			
H4A—O4—H4B	111 (3)	C11—C10—H10	119.4
O1—N1—O2	123.34 (19)	C9—C10—H10	119.4
O1—N1—C3	118.57 (18)	C10—C11—C12	121.19 (19)
O2—N1—C3	118.09 (18)	C10—C11—H11	119.4
C7—N2—N3	118.26 (17)	C12—C11—H11	119.4
C7—N2—H2N	122.7 (15)	N4—C12—C13	120.59 (18)
N3—N2—H2N	118.4 (15)	N4—C12—C11	121.96 (19)
C8—N3—N2	113.98 (17)	C13—C12—C11	117.45 (18)
C12—N4—C15	122.27 (17)	C14—C13—C12	120.52 (19)
C12—N4—C17	121.30 (18)	C14—C13—H13	119.7
C15—N4—C17	116.15 (17)	C12—C13—H13	119.7
C2—C1—C6	120.75 (19)	C13—C14—C9	122.1 (2)
C2—C1—H1	119.6	C13—C14—H14	118.9
C6—C1—H1	119.6	C9—C14—H14	118.9
C3—C2—C1	118.25 (19)	N4—C15—C16	114.2 (2)
C3—C2—H2	120.9	N4—C15—H15A	108.7
C1—C2—H2	120.9	C16—C15—H15A	108.7
C4—C3—C2	122.61 (19)	N4—C15—H15B	108.7
C4—C3—N1	118.86 (19)	C16—C15—H15B	108.7
C2—C3—N1	118.52 (18)	H15A—C15—H15B	107.6
C3—C4—C5	118.47 (19)	C15—C16—H16A	109.5
C3—C4—H4	120.8	C15—C16—H16B	109.5
C5—C4—H4	120.8	H16A—C16—H16B	109.5
C6—C5—C4	120.35 (18)	C15—C16—H16C	109.5
C6—C5—H5	119.8	H16A—C16—H16C	109.5
C4—C5—H5	119.8	H16B—C16—H16C	109.5
C5—C6—C1	119.55 (18)	N4—C17—C18	113.5 (2)
C5—C6—C7	123.55 (17)	N4—C17—H17A	108.9
C1—C6—C7	116.88 (18)	C18—C17—H17A	108.9
O3—C7—N2	122.99 (18)	N4—C17—H17B	108.9
O3—C7—C6	120.63 (17)	C18—C17—H17B	108.9
N2—C7—C6	116.38 (17)	H17A—C17—H17B	107.7
N3—C8—C9	122.62 (19)	C17—C18—H18A	109.5
N3—C8—H8	118.7	C17—C18—H18B	109.5
C9—C8—H8	118.7	H18A—C18—H18B	109.5
C10—C9—C14	117.46 (18)	C17—C18—H18C	109.5
C10—C9—C8	123.73 (18)	H18A—C18—H18C	109.5
C14—C9—C8	118.80 (19)	H18B—C18—H18C	109.5
C11—C10—C9	121.26 (18)		
			
C7—N2—N3—C8	−174.7 (2)	N2—N3—C8—C9	−178.3 (2)
C6—C1—C2—C3	−0.7 (4)	N3—C8—C9—C10	6.9 (3)
C1—C2—C3—C4	0.6 (4)	N3—C8—C9—C14	−174.4 (2)
C1—C2—C3—N1	−178.9 (2)	C14—C9—C10—C11	0.8 (3)
O1—N1—C3—C4	176.0 (2)	C8—C9—C10—C11	179.6 (2)
O2—N1—C3—C4	−4.6 (3)	C9—C10—C11—C12	0.2 (3)
O1—N1—C3—C2	−4.4 (3)	C15—N4—C12—C13	167.7 (2)
O2—N1—C3—C2	175.0 (2)	C17—N4—C12—C13	−18.6 (3)
C2—C3—C4—C5	−0.2 (4)	C15—N4—C12—C11	−12.3 (3)
N1—C3—C4—C5	179.4 (2)	C17—N4—C12—C11	161.3 (2)
C3—C4—C5—C6	−0.2 (3)	C10—C11—C12—N4	179.1 (2)
C4—C5—C6—C1	0.1 (3)	C10—C11—C12—C13	−1.0 (3)
C4—C5—C6—C7	−178.5 (2)	N4—C12—C13—C14	−179.4 (2)
C2—C1—C6—C5	0.3 (3)	C11—C12—C13—C14	0.6 (3)
C2—C1—C6—C7	179.1 (2)	C12—C13—C14—C9	0.5 (4)
N3—N2—C7—O3	0.1 (3)	C10—C9—C14—C13	−1.2 (3)
N3—N2—C7—C6	−179.4 (2)	C8—C9—C14—C13	179.9 (2)
C5—C6—C7—O3	160.9 (2)	C12—N4—C15—C16	91.3 (3)
C1—C6—C7—O3	−17.8 (3)	C17—N4—C15—C16	−82.7 (3)
C5—C6—C7—N2	−19.6 (3)	C12—N4—C17—C18	93.1 (3)
C1—C6—C7—N2	161.7 (2)	C15—N4—C17—C18	−92.8 (3)

### Hydrogen-bond geometry (Å, °)

**Table:**

*D*—H···*A*	*D*—H	H···*A*	*D*···*A*	*D*—H···*A*
O4—H4B···O3^i^	0.83 (4)	2.23 (4)	3.009 (3)	156 (3)
N2—H2N···O4	0.88 (2)	2.00 (2)	2.861 (2)	165 (2)
O4—H4A···O3^ii^	0.85 (3)	2.06 (4)	2.823 (2)	149 (3)
O4—H4A···N3^ii^	0.85 (3)	2.57 (3)	3.250 (3)	138 (3)
C5—H5···O3^ii^	0.95	2.54	3.308 (2)	138
C8—H8···O4	0.95	2.50	3.270 (3)	138
C13—H13···O2^iii^	0.95	2.51	3.350 (3)	148

Symmetry codes: (i) −*x*+1/2, −*y*+3/2, −*z*+1; (ii) *x*, −*y*+1, *z*−1/2; (iii) −*x*+1/2, *y*−1/2, −*z*+1/2.
